# Wireless Computer-Supported Cooperative Work: A Pilot Experiment on Art and Brain–Computer Interfaces

**DOI:** 10.3390/brainsci9040094

**Published:** 2019-04-25

**Authors:** Gabriel G. De la Torre, Sara Gonzalez-Torre, Carlos Muñoz, Manuel A. Garcia

**Affiliations:** 1 Department of Psychology, University of Cadiz, Campus Rio San Pedro 11510, Puerto Real (Cádiz) Spain; sara.gtm@uca.es (S.G.-T.); Mantonio.garcia@uca.es (M.A.G.); 2 Engineering Superior College, University of Cadiz, Cádiz 11519, Spain; carl16011992@gmail.com

**Keywords:** BCI, computer-supported cooperative work, neuropsychology, arts

## Abstract

The present case study looked into the feasibility of using brain–computer interface (BCI) technology combined with computer-supported cooperative work (CSCW) in a wireless network. We had two objectives; first, to test the wireless BCI-based configuration and the practical use of this idea we assessed workload perception in participants located several kilometers apart taking part in the same drawing task. Second, we studied the cortical activation patterns of participants performing the drawing task with and without the BCI technology. Results showed higher mental workload perception and broader cortical activation (frontal-temporal-occipital) under BCI experimental conditions. This idea shows a possible application of BCI research in the social field, where two or more users could engage in a computer networking task using BCI technology over the internet. New research avenues for CSCW are discussed and possibilities for future research are given.

## 1. Introduction

Brain–computer interfaces (BCIs) enable users to send commands to a computer using brain activity alone. This technology forms part of a rapidly growing interdisciplinary field of research encompassing neuroscience, computer science, engineering, and, more recently, psychology. BCI devices can be utilized in several areas of research, including physical rehabilitation, neurological disorders, human–machine interactions, and computer-supported cooperative work (CSCW). BCIs enable two individuals to connect to one another through an Internet network to perform simple computer-based cooperative tasks. While these tasks may help in creating a new mental state or shared consciousness, they can have high perceived workload demands.

Context-aware CSCW interactions provide relevant information or services to the user while they are carrying out the task. Sohlenkamp [1] suggested that awareness in the context of CSCW enables users to coordinate and structure their work. This can be used to facilitate collaborative work [2]. In the context of CSCW systems, a group can be seen as a number of individuals who interact directly or through shared devices who perceive themselves as a group [3]. Group awareness can be broadly defined as the consciousness and knowledge of various aspects of the group and its members. 

Research on the interaction between computers and internal states of the human body spans several disciplines, including cognitive psychology, neuroscience, affective and physiological computing, and human–computer interaction (HCI). However, most of the BCI system and physiology-based interaction studies published to date have used a single user, with the possibility of their use in collaborative scenarios or CSCW remaining unexplored. This study aimed to develop a multi-user network system using electroencephalography (EEG)-based BCIs with an online whiteboard-based drawing tool for collaborative art production.

There are three different types of BCI that are currently available: invasive, partially invasive, and non-invasive [4]. Invasive BCIs record signals from electrodes implanted surgically over the cortex. Partially invasive BCIs are implanted inside the skull but rest outside of the brain. Non-invasive BCIs record brain activity from an external electrode cap or headset with EEG sensors. The EEG based system is the most-studied non-invasive interface, mainly due to its portability, ease of use, and low set-up cost [5]. BCIs are primarily employed in HCI studies to detect brain patterns. In order to manipulate elements directly with the brain, we added the artistic component to our task so spontaneous creativity and global or group awareness could arise from two connected minds, using the BCI, in the same CSCW network. The study had two goals: first, to test whether two people connected using a BCI on the Internet could perform an artistic task with a collaborative Windows®-based application; second, to investigate the perceived task demand, alongside EEG patterns obtained during the task. 

## 2. Methods

### 2.1. Preparation of the Experiment

**Figure 1:**
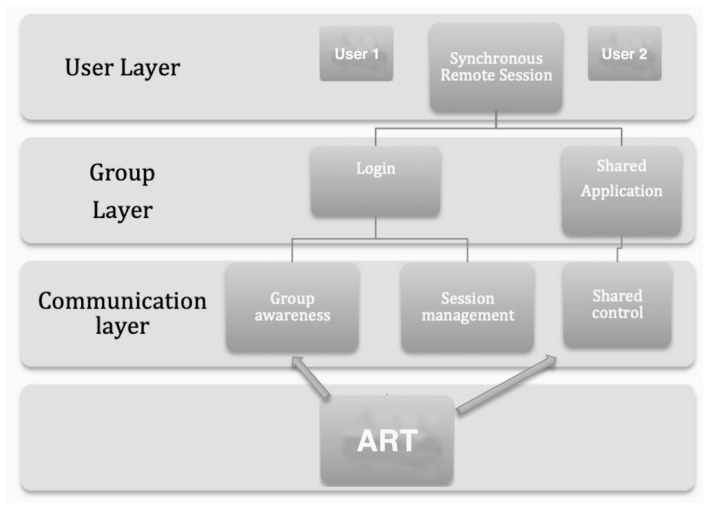
Brain–computer interface (BCI) computer-supported cooperative work (CSCW) architecture.

Participants in this experiment used an Emotiv Epoc+ headset to control a collaborative online painting application. Two participants (female and male, both aged 25) in different locations joined the online collaborative painting application *FlockDraw*—an example of computer-supported cooperative work (CSCW). Once they joined the synchronous remote session with this application, all actions were carried out using the BCI headset. Figure 1 shows the basic CSCW architecture of the system with three main layers (user, group, and communication) during the experiment using the BCI and the resulting cooperative ART or drawing.

**Figure 2:**
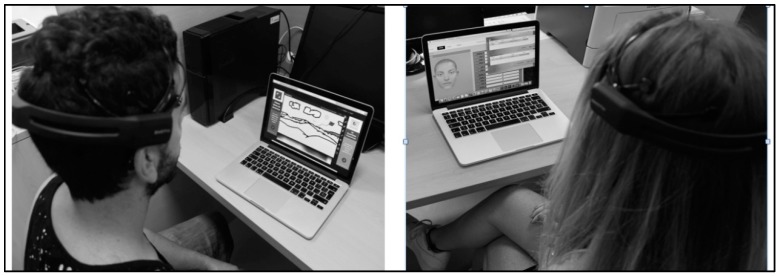
Participants wearing BCI headsets in front of computers during the experiment.

The Emotiv Epoc+ wireless headset has 14 wet sensors and 2 reference sensors, enabling EEG and facial expression data to be recorded with optimal positioning and accurate spatial resolution [6]. An integrated gyroscope generates positional information for cursor and camera controls, connected wirelessly through a USB connection. The sixteen sensors are placed on the international 10–20 system [7] —an internationally recognized method which describes the electrode placement on the scalp for EEG tests or experiments. The system relies upon a combination of cognitive and facial/muscular recordings which can provide inputs to a computer [4]. The gyroscope embedded in the Emotiv Epoc+ headset provides information about head movements through a speed signal. This feature was used to control the mouse/pen on the screen during the drawing task [8]. The Emotiv Epoc+ headset is used in combination with the Emotiv *TestBench* control panel, where new users can be created and trained (Figure 2). Emotiv Epoc+ uses the user profile to personalize the training routine and to map users’ brain patterns. In a training session lasting no more than 1 hour, users’ skills increased approximately up to 45% for the up and down movements, and by around 10% for left and right. 

Training the profile requires practice, especially when the user needs to train two or more actions. The EmoKey application was used connected with Emotiv Control Panel to generate action processes for each identified thought or brain activation pattern. The EmoKey application then transfers these action processes to a client/server application, with each process being treated as a drawing action through a socket connection to the online drawing application. 

*Flockdraw* is an online whiteboard-based painting and drawing tool allowing an unlimited number of people to draw simultaneously on the same virtual surface. For this study, we used the NASA task load index (NASA-TLX) to measure perceived workload. This is a multi-dimensional rating procedure with six subscales: mental demands, physical demands, temporal demands, performance, effort, and frustration [9]. The overall workload score was calculated as in Felton, Williams, Vanderheiden & Radwin [10].

### 2.2. Experiment

EEG patterns were recorded when the participants were drawing with the BCI, and with a computer mouse instead of the BCI. Two new participants (2 females, age 18) completed separate drawing tasks using the Emotiv Epoc+ system and *Flockdraw* online application. During the task, participants sat in front of a laptop wearing the headset and were instructed to remain still.

### 2.3. Signal Acquisition and Processing

EEG data were acquired using a portable EEG Neuro-headset or the Emotiv Epoc+. The Emotiv Epoc+ can record neural signals generated in response to distinct subject actions using its 14 assembly electrode sensors (i.e., AF3, F7, F3, FC5, T7, P7, O1, O2, P8, T8, FC6, F4, F8, AF4)_ and 2 reference electrodes (i.e., P3 and P4). The acquired EEG signal was transmitted to EEGLAB [11], where it was further processed. The EEG dataset was recorded at a sampling frequency of 128 Hz and pre-filtered using the basic EEGLAB FIR filter option. EEGLAB v 15.0.6 for MATLAB was used for the signal processing of real time EEG data. The independent component analysis (ICA) algorithm of EEGLAB was used to detect eye blinks. The ICA decomposition of signals leads to the extraction of maximally temporally independent EEG signals corresponding to any activity present in the channel data. ICA decomposition with the algorithm *runica.m* (selects neural components having super-Gaussian activity distribution), available in the EEGLAB toolbox, was implemented for a sample of 10 seconds in order to extract time–frequency transforms and data statistics. Spectral and clustering analysis was performed to extract the frequency component of the EEG signal. Brain components were generated by applying ICA and CSP/DSP to EEG signals. Further analysis of independent components (ICs) was performed following the same direction as previous studies [12]. ICs with bilaterally distributed scalp maps were fitted with a dual equivalent dipole model. ICs were then clustered using a K-means clustering algorithm applied to the matrix of IC pairs using EEGLAB. EEG data from each painting task were recorded, and representative EEG epochs of 1000 ms (200 ms baseline) were extracted off-line for each condition. EEG epochs from each channel were baseline corrected by subtracting the average activity of that channel during the baseline period. Epochs were then re-referenced to linked-mastoid electrodes. Ocular artifacts were corrected with an eye-movement correction algorithm. AC artifacts were removed using the clean-line plugin for EEGLAB. 

## 3. Results

**Figure 3:**
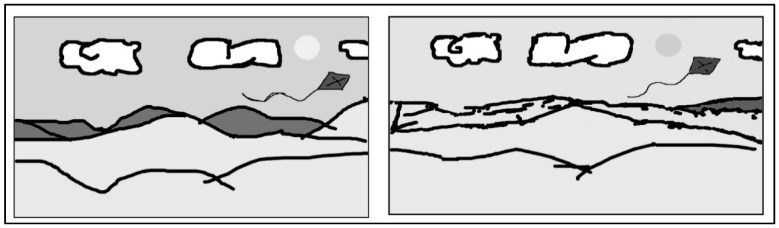
Painting performed in *FlockDraw* collaborative online software without BCI (**left**) and with BCI (**right**).

The group of participants was too small for statistical analysis, and therefore only some individual data are reported descriptively. The main purpose of the experiment was to test the feasibility of a cooperative network/BCI-based drawing task. The information transfer rate is an important aspect in BCI experimentation. In this case, it was calculated to measure the speed command selection (right, left, up, down, center, color selection). The most-used method for information transfer rate calculation is the approach suggested by Wolpaw [13]. Generally, this method assumes the specific accuracy for each selection and the probability of each undesired selection to be the same. In the case of this brain painting task application, these criteria are difficult to meet [14,15]. Therefore, the formula should be interpreted as an approximation of the information transfer rate [16]. The system was tested on healthy participants with an information transfer rate of 2 bits and accuracy higher than 90%. The mean time for the BCI drawing task was 1350.5 seconds (SD: 449.75 s) and 232.5 seconds (SD: 91.5 s) for no BCI drawing. Although the drawing obtained by both participants was relatively simple, this demonstrates the first case of creating art by the brain activity of two different minds connected and spontaneously collaborating through the Internet (Figure 3). Based on the subjective data obtained, the task had high mental and physical demands, as was particularly evident for mental demand, effort, and frustration indices (Table 1). 

**Figure 4:**
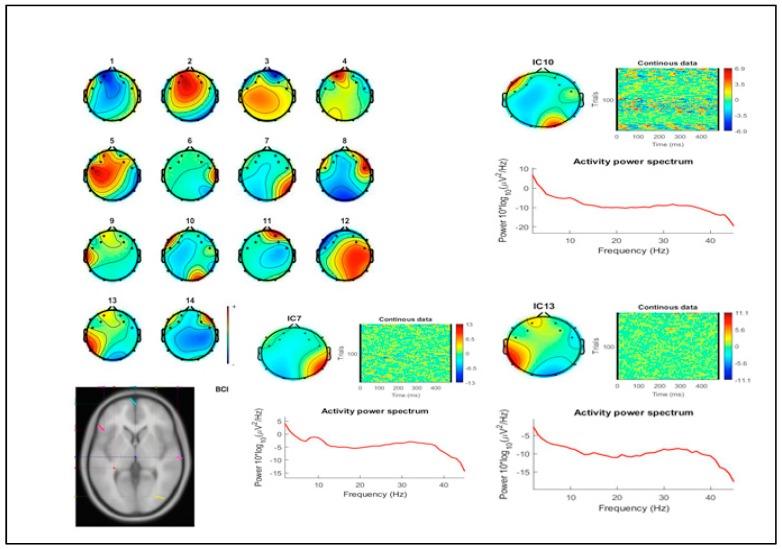
Scalp maps, spectral, DIPFIT, and posterior independent components (ICs) (scalp map and power spectra) for BCI condition. Top axial Magnetic Resonance (MR) view (**bottom left**) shows the results of modeling the grand mean scalp maps for each of the significant IC clusters as the projection of an equivalent dipole using the DIPFIT tool box for EEGLAB.

**Figure 5:**
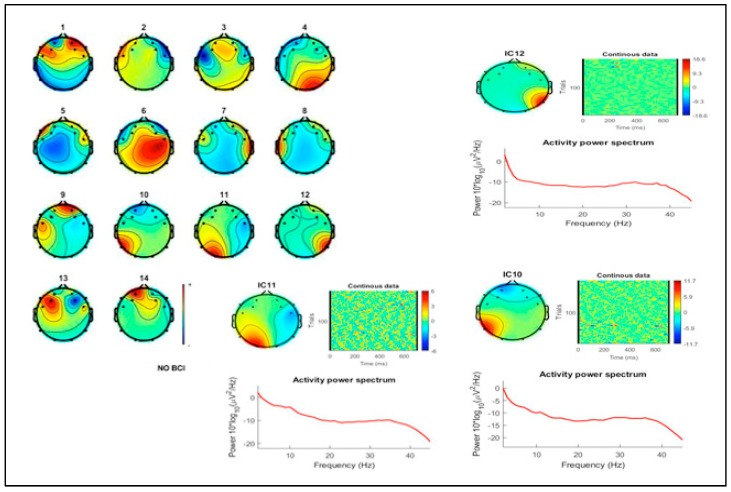
Scalp maps, power spectra, and posterior ICs for NO-BCI condition.

Despite the small group of participants in this case study, some EEG activity data are reported for reference. Results for the analysis of EEG activity showed a greater involvement of cortical load in the BCI painting task compared to the no BCI condition. Spectral analysis showed greater general and occipital activation during the BCI painting task. This activation was clearly increased during the BCI task for frequencies in the 20–35 Hz range, as shown in the ICs matrix for both conditions (Figure 4 and Figure 5). Although the activation patterns acquired with the EEG BCI headset showed a clear fronto-temporo-occipital distribution, this was likely due to changes in activity in occipital regions as they are involved in visual perception and processing. 

Finally, participants completed NASA-TLX. Differences between BCI and non-BCI situations were clear, with higher scores for the effort index and the physical demand alongside time and mental demand (Table 2). It is important to note that participant 2 scored 0 in mental demand for the BCI task because she said she had fun doing the task, although her effort score was the highest of both participants. It is possible that the enjoyable nature of the task reduces the perceived mental workload, as has been suggested to be the case for other BCIs, in virtual reality and drone control situations [17].

## 4. Discussion

Mental workload is an important consideration when designing tasks or procedures that require user attention. In this study, mental workload was recorded to investigate the feasibility of BCIs for assistive technology and alternative control in CSCW or multi-mind connected network scenarios, and to make comparisons between different computer input modalities. We also did some preliminary brain activation patterns using a BCI device able to record EEG data. Many studies have evaluated strategies to reduce mental effort when performing computer tasks [18] which tend to require high visual and cognitive demands, leading to an increase in mental workload [19]. In this study, participants several kilometers apart were able to create a simple painting using EEG patterns transmitted via connected BCI systems. This novel step into mental networks connected through these devices opens a new avenue for CSCW. We found that drawing with a BCI required greater mental workload and effort in comparison to a conventional physical, approach. Levels of frustration were also substantially increased when drawing under BCI conditions. It is clear from these results that the technology needs to be improved and a better understanding of the human brain and its cognitive processing is required. 

The final drawings obtained from the participants did not differ greatly in comparison to when participants were not using the BCI system; although it required greater effort, they were able to achieve similar results. Mental workload could be a helpful measure when designing these technological improvements, and the NASA-TLX was a sensitive measure of this. This increased effort was also evidenced by greater activation in brain regions responsible for visual perception (occipital regions) and executive functioning (frontal cortices). Previous studies have suggested a role of the P300 in ERP (event-related potential) BCI applications, with this being altered in both healthy subjects and patients with amyotrophic lateral sclerosis (ALS) [15,20,21] during BCI tasks, including those requiring drawing [22,23,24,25]. We are not aware of any other published study investigating the neural activity associated with CSCW with BCI in drawing or other tasks. In our task we observed greater occipital involvement during BCI than during a normal drawing task. It has previously been suggested that actions themselves form part of the perceptual process [26]; this is in accordance with the greater activation of regions associated with visual processing during the BCI drawing task. However, BCI systems have some limitations, especially for this type of psychosocial experiment. Among BCI limitations we can mention the large amount of training data required from users and the relatively long times required to calibrate classifiers. Some methods have been suggested to help with these limitations, including SBLaplace algorithms to obtain better overall classification performance for ERP-based BCIs (especially for small sample size scenarios), improving the practicability of BCI systems [27]. Additionally, a potential limitation of motor-imagery-based BCIs is the requirement of relatively long times to record sufficient EEG data for robust classifier training [28]. Flexible group sparse discriminative analysis algorithms based on Moreau-Yosida regularization have been proposed for alleviating the common undersampling problem present with small training samples in ERP, especially P-300 BCI-based studies [29]. 

There were several limitations in particular to our study. Only a small number of participants were used, although it is difficult to use a large number of participants in these experiments. Future research may connect multiple individuals with BCIs. Secondly, the Emotiv Epoc+ BCI system has only 14 channels and is not intended to record or analyze EEG or ERP activity. However, this pilot study was conducted with the view that similar studies conducted in the future may utilize more accurate and standardized setups as technology evolves. Another problem to be solved is possible network delay/bandwidth issues that may occur, affecting the interactions.

Drawing with BCI systems may improve the quality of life for patients with severe motor disabilities. This experiment also demonstrates a novel form of CSCW where several participants can participate in a common process or task and produce a result that is the sum of their different perceptions, intentions, and consciousness. In the future, computer processes and associated events may occur as a result of single minds converging to allow this to happen.

This pilot study demonstrates the possible use of BCI devices for CSCW, but also raises some limitations of their use. The high mental workload demands reported by participants demonstrate the need for the further development of the ergonomics, usability, and software integration of this technology. The NASA-TLX was able to detect the increased workload required for the BCI drawing task. EEG recordings also showed increased general and occipital activation during the BCI during the task. Further research is required using BCIs and multiple participants, networks, and paradigms. Further research should be done in other domains, such as computer programing, mathematics, problem solving, or language.

## 5. Conclusions

This case study tested the feasibility of a BCI setup for CSCW task over the Internet. Several limitations were detected but in general the application was possible. NASA-TLX detected subjective increased perceived workload demand in BCI condition compared to no BCI task. This idea opens new paths for application of BCI in the social field, where two or more users could engage in networking tasks using Internet based applications. The possibilities are especially interesting for research with patients with severe motor problems. 

## Figures and Tables

**Table 1:** NASA task load index (NASA-TLX) results for the BCI/CSCW painting task (non-BCI/CSCW).

Participant	Global Score	Mental Demand	Physical Demand	Time Demand	Performance	Effort	Frustration Level
Participant 1 male	65.86 (2.66)	77 (9)	62 (0)	13 (2)	15 (1)	93 (0)	67 (1)
Participant 2 female	68.81 16.13)	84 (18)	52(3)	1 (3)	49 (28)	71 (9)	71 (4)
Mean	67.33 (9.39)	80.50 (13.50)	57 (1.50)	7 (2.50)	32 (14.50)	82 (4.50)	69 (2.50)
*SD*	2.08 (9.52)	4.94 (6.36)	7.07 (2.12)	8.48 (.70)	24.04 (19.09)	15.55(6.36)	2.82 (2.12)

**Table 2:** NASA-TLX results for participants in experiment part B (painting task BCI and NO BCI, NO CSCW *).

Participant	Global Score	Mental Demand	Physical Demand	Time Demand	Performance	Effort	Frustration Level
	**BCI**						
Participant 1	47.80	72	50	83	28	39	43
Participant 2	40.86	0	67	5	26	74	25
Mean (SD)	44.33 (4.90)	36 (50.91)	58.50 (12.02)	44 (55.15)	27 (1.41)	56.50 (24.70)	34 (12.70)
	**NO BCI**						
Participant 1	35.92	11	10	7	55	41	33
Participant 2	5.60	12	1	2	5	1	3
Mean (SD)	20.76 (21.43)	11.50 (.70)	5.50 (4.50)	4.50 (2.50)	30(35.35)	21 (28.28)	18(21.21)

* CSCW: computer-supported cooperative work. BCI: Brain Computer Interface.
